# Report to the Erie (Pennsylvania) County Medical Society, on the Prevalence of Suits for Mal-Practice

**Published:** 1850-02

**Authors:** 


*Report to the Erie (Pennsylvania) County Medical Society, on the prevalence of Suits for Mal-Practice.*

We find the following report in the columns of the Erie (Penn.) Gazette. The Chairman of the Committee, Dr. Wood, Surgeon U. S. Navy, is the author of an interesting paper on the same subject, communicated for the American Journal of Medical Sciences, and contained in Eclectic Department of the January aumber of this Journal. The subject is an important one, more especially to all who practice surgery, and we suggest that, with a view to excite attention to it, and to give a proper direction to the reflections of the public, it would be well for members of the profession in different places, to procure the insertion of this report in periodicals having a general circulation. It is due to the public, as well as to ourselves, that we employ proper means to diffuse information on this, as well as other subjects in which the interests of both are alike involved.—Ed. Buffalo Med. Journal.

The following Report was read before the Erie County Medical Society at its last meeting:

The Committee to which was referred the subject of suits for Mal-Practice, report: That those practitioners who are known to the profession and to the community, as being the best qualified among us, are frequently arraigned before the Courts on such suits, and often unjustly convicted of the charge, while those known as gross and ignorant pretenders, whose whole existence is a continued system of mal-practice, pass unnoticed and unharmed.

These suits appear in the majority of cases, to be instigated by the worst motives, and are terminated by decisions contrary to truth, justice, and common sense.

Such results are undoubtedly a perversion of the legal principles and provisions under which mal-practice suits are instituted, and the Committee will now enpeavor to set forth the influences to which the evil is to be attributed.

The principal and fountain source of all the wrong is the want of popular information as to the nature of medical science, and as to what should in all fairness be expected from a qualified physician and surgeon.

From this prevailing ignorance and misconception, the medical practitioner is expected to be the bold controller of nature, instead of her vigilant observer, faithful follower and intelligent assistant. Certain and mechanical results are confidently looked for, where true and high science would teach that nothing but probabilities can be reached by human skill. It is true that correct scientific or professional knowledge increases those probabilities, but those who study our profession most laboriously and profoundly know that their chances of arresting disease depend upon the extent and variety of their acquirements, but that nothing can give the power of certainty. Hence the conscientious practitioner of medicine feels that there can be no limit to the obligation and the necessity for adding to his attainments, and that his duty is to go from one acquirement to another. One so enlightened will promise no certain cure for the slightest ailment, for he well knows that under the mysterious agency of vital laws which are hidden by Providence from the scrutiny of man, death may result from the slightest derangement of the human organism. A scratch may terminate in fatal mortification; tetanus and death be caused by the extraction of a tooth, or even a too close clipping of the nails, and in fifteen minutes life has been terminated by the sting of a wasp. Hence the well informed physician, though he may be confident of his ability to select with judgment among the various remedies with which he is familiar, and though he may know that they are rationally suited to the case he is treating, awaits with cautious hope rather than bold assurance a result which it is not with him to determine. But because an infallible certainty cannot be reached, there is no reason or excuse for failure in the labors of science; the professional man is bound by every increased chance and probability to go on. The uncertainty which attends our profession is applicable to every avocation which is connected with the laws of nature. All such professions must acknowledge the same subjection to influences beyond their control, as attaches to that of him who deals with the living, thinking human being, and must submit all their hopes in contingencies which they can neither forsee nor prevent.

The agriculturalist who, with a practical knowledge of his pursuit, has an intelligent acquaintance with the principles which influence it—with the control of climates and seasons, and with the nature of soils—has a much greater chance of success than one who works in blindness and ignorance, and during a series of years his average returns will be much greater and more secure; but with the application of all his knowledge and skill, he cannot on any one year feel an assurance of success. Droughts may parch his fields or floods drown them, frosts nip his fruits in the bud, or blight his grain in the ear.

The navigator carefully studies the currents which sweep noiselessly through the ocean, and the laws which govern the winds blowing over its surface, and when his vessel is trusted to this vast machinery of nature, it is in no blind ignorance of the forces moving it. The addition made to the stores of nautical knowledge in all its branches, has not only shortened the time of long voyages, but added to the total average security with which the ocean is traversed, and it is clear that the probabilities of success will be greater for the vessel in proportion to the intelligence and professional acquirements of those having her in charge; but the finest ship that ever floated, navigated with all the skill which man's intellect can display, may never reach her port—may be cast, a shattered wreck, on the shore, and even if she be brought into harbor, it may be dismantled, rigging, spars and cargo all gône, and yet, in this crippled condition, she may be a monument of more nautical science than if she had made her voyage unharmed.

The foregoing considerations apply with yet greater force to our profession,, which deals not only with nature and its physical laws, but with the moral and intellectual nature of man; and this wonderful combination of average and increasing success with an ever existing uncertainty, is a beautiful exhibition of the harmonious laws by which the wisdom of Providence reconciles apparent contrariety. A general and progressive success is necessary to stimulate man to steady and progressive exertion, but did he reach certainly where the laws of nature are concerned, he would lose his dependence upon God, and hence occasionally all the arrangements and protections of science and philosophy vanish before the asserted omnipotence of the Deity, and yet man has no excuse to refrain from further study and renewed exertion.

It is an ignorance of, or want of reflection upon, these principles which forms the foundation for the prevalence of quackery, and of the unjust persecutions which pursue the regular proctitioner, and display themselves in groundless suits. The pretender to medicine meets the popular expectation by promising infallible remedies for every disease. The quack is not always, nor indeed generally, an impostor; he partakes of the popular ignorance, and the popular expectation, and promises infallibility because he believes infallibility to be a possibility; and ignorant of the labcrious processes of scientific induction, believes that one man can jump at the desideratum as well as another, and hence boldly sets up his own pretensions.

The reasonable expectations of professional usefulness having been lost sight of, the simple common sense means—those in which cause and effect bear an apparent relation—are set aside also, and to accomplish the expected wonders, wonderful means are brought in—those whose mode of action are as incomprehensible as their expected effects are inconsistent with the teachings of nature and the designs of Providence. The wonderful and the marvellous—those which overturn the judgment and set up the imagination—are appealed to, and we have the whole tribe of quacks flooding the community with magic pills and pain extractors, charms, amulets, and the blood of black cats—with mystical vials and infinitesimal doses, the ghost of a remedy in search often of but the ghost of a disease.*

In such a state of things, the reasonable means pointed out by correct knowledge, become in the popular eye, the proof of mal-practice; and a regular practitioner who has been so unfortunate as to obey nature and truth, is hunted as the victim of popular prejudice, while the quack who has complied with that prejudice goes free. We have the testimony of a highly intelligent and experienced judge, that in all his service on the bench, he has known only one instance in which a suit for mal-practice was against a quack doctor, but he had known many against regular physicians. Sympathy naturally attaches to the victim of incurable disease or injury; but from the state of feeling we have endeavored to analyse, the condition

---

*Note.—This expression is not used as a mere figure, or an exaggeration of ridicule, but as the statement of a fact. Hahnemann denies the materiality of disease. He says: "Diseases are not, and cannot, be mechanical or chemical changes of the material substance of the body; they do not depend on a morbid material principle," and again, "Diseases cannot cease to be *dynamic alterations that our spiritual life feels!*" In this he is consistent with the use of immaterial remedies. Ridicule is no proper method of addressing prejudice and error, however absurd they may be, and it may be used with as much efficiency against truth as against error. We should always bear in mind that what may appear absurd to a person possessed of a certain kind of information, may appear perfectly rational to all destitute of that kind of information, and hence the fact of their belief is no imputation upon either their judgment or honesty. If any are desirous of correcting such errors in their fellow men, it should be by imparting, if possible, the information which leads to a correct conclusion. This cannot in all cases be done. The mode and means of judging, acquired by years of mental labor in a special profession, cannot be imparted in a single day, a single conversation, a single volume. The whole years of education must be gone through with to reach the same capacity of judging, and hence the opinion of a profession is entitled to faith and respect even though the reasons by which the opinion is reached may not be apparent to those of the profession. Reason teaches this. Mental labor consists not only in acquiring, but in much discipline to teach the faculties how to acquire. It is unreasonable then to expect that those who have by severe mental discipline taught themselves the power of acquiring, or that those who, having this discipline, have not acquired the facts of a certain profession, should yet reach as correct conclusions as those who have been devoted both to the means and the matter of that profession. If these things are not correct, then is all mental labor and professional pursuit a waste of time; and, being correct, it would certainly seem like great arrogance and self-conceit for individuals not of a profession, to set up and boldly maintain dogmas and opinions contrary to the verdict and decisions of that profession. But it is evident that such things are done in all honesty and faith by those who are not either arrogant or self-conceited. What then is the influence, placing them in such a false position? We think this influence can be shown in a popular exposition of the nature of medical evidence, medical systems, and the vast arrangement of moral and mechanical medical machinery. The subjects are interesting, and if the editors of the *Gazette* will trust to our handling them, and favor us with a little space, we will, in a subsequent article, make an endeavor to answer the above question. W. M. W.

of the disease or injury is ever attributed to the practitioner having the case in charge, and not to laws beyond his control.

It is easy now to trace the influences which bring the case into Court. The quacks have all their interests and their feelings concerned in bringing discredit upon the regular physician; they act upon the popular prejudices, and foment the suit. The sympathy to which we have alluded, misdirected, calls for vengeance; and added to these influences is the cupidity of those who would escape the payment of their bill, or desire to make a market of their misfortunes.

The popular expectation, the erroneous misconception, the natural sympathy for the patient; and the general prejudice against the practitioner take their seats in the jury box. No discrimination selects the medical witnesses; those who have instigated the suit, and men who neither understand the meaning nor the pronunciation of the terms they use, stand on the same levil with the qualified practitioner, and from the influences we have mentioned, the chances are that their testimony is the most influential.

The jury is, perhaps, told that as the work of the mechanic is rejected, unless it comes up to a standard of perfection, so the work of the physician and surgeon must come up to a like perfection, and such illustrations are received as parallel and analogical. By such reasoning, the mysteries of vitality, of that machine fearfully and wonderfully made in the image of its Maker, and living by the breath of the Deity, is reduced to a level with inanimate wood, stone, and iron, obedient to the mechanic's hand.

But what is to be the measure of the perfection of the physician's art, or of that of the surgeon? It may be only in his power to save life by the loss of limbs, to save limbs by preserving them with deformity. The popular eye can see how much has been lost, but only science can see how much has been saved.

Under such circumstances, it is not surprising that practitioners should be convicted, as we know they have been, upon a point of anatomy which has no existence, and upon modes of treatment which a jury of professional men would have pronounced correct, judicious, and scientific.

For all this ignorance, misconception and errors, a fearful retribution is being visited upon the community. No matter how highly qualified a physician and surgeon may be, he must, in self defence, refuse the responsibilities of cases involving the contingency of a suit for mal-practice. Some of the most skillful men of our country, now refuse such cases without their pay beforehand, and a guarantee against a mal-practice suit. It is not in the power of all to comply with such terms, and yet none can yield to the claims of humanity without risks which few dare encounter.

No man may imagine himself safe from the whole train of evils resulting from this state of affairs. They impose upon the entire community the severest penalty which mortality can pay; they cost the nation half its life, and consequently half its entire happiness and usefulness. With all the elements of health and long life in our country, statistics—figures—lead to the fearful inference that our average length of life is but little if any more than half that enjoyed by over-crowded, over-worked, vicious and half-starved Europe, and the main cause of the disparity is to be found in the difference that there the care of health and life is trusted, by every precaution, to those qualified, by suitable education, and here every ignorant and conceited pretender is permitted to assume the solemn responsibilities of managing the health of his fellow citizens, and making their lives the playthings of his blind folly and vain presumption.

We infer that the dawn of a better day is at hand, and that the people will be aroused by the sense of self-preservation, to take the necessary steps for securing competent qualifications in practitioners of medicine.

The Governor of the State of New York, in his late message, has pnt his hand to the work. We quote the following from the earlier paragraphs of the message:

"No subject more universally affects all classes, and all members of the community, than that of the public health. I therefore earnestly request your attention to the existing laws on this subject, and suggest the propriety of their careful review and amendment, especially with a view to secure the benefit of the combined experience of scientific and learned men throughout the State, with respect to the origin, the causes, the progress, and the tteatment of all malignant or infectious diseases.

"It may also be well to consider whether the time has not arrived, when the State is called upon to contribute its aid more efficiently than it has hitherto done, to advance the cause of Medical Education. Every inhabitant of the State, at some time or other, feels the need of the physician, and is interested that he should be learned and skilful."

It is more easy to point out the causes of, than the remedy for, these evils, and more easy to name the remedies than to say how they shall be carried into effectual operation.

The chief remedy would be the diffusion among the people, of correct ideas in relation to the duties and obligations of the profession, and upon this subject it should be borne in mind that the standard of qualification should be in some degree correspondent to the locality. It cannot be expected that the hard worked, and poorly paid country practitioner should have the same qualifications as those partaking the facilities and rewards of the large cities. The country must not expect a degree of service beyond its own ability to reward. No matter how much ability may be possessed by the practitioner, individually, he must be indebted to favoring circumstances for the highest qualification. It is not to be expected that the surgeon, who sees but half a dozen surgical cases in the course of a lifetime, can display the same skill as one, who, perhaps, at the head of a large hospital, has that many cases every day.

Again, none should be admitted on the witness stand but those who have some evidence of fair qualifications as medical men, and finally the protection of the profession and of the people rests much with the President, and Judges of the Courts. By instructing the juries simply as to what they have any right to demand, many an unjust verdict would be arrested.

It is believed that with this just care on the part of President Judges of the Courts, and a proper discrimination in regard to witnesses, much would be done to give a just direction to the law, and to ensure that verdicts and penalties in cases of mal-practice would be only against those who had failed in the lawful and reasonable acquirements which they should possess, or attentions which they should bestow.

The report is respectfully submitted, WM. MAXWELL WOOD,
A. BEEBE,
S. DICKINSON.

